# Child occupant injuries in the United Arab Emirates: a case for a safe system approach

**DOI:** 10.3389/fpubh.2026.1844331

**Published:** 2026-09-25

**Authors:** Muhammad Uba Abdulazeez, Aminu S. Abdullahi, Sjaan Koppel, Michal Grivna, Kassim Abdullah

**Affiliations:** 1 Emirates Centre for Mobility Research, United Arab Emirates University, Al Ain, United Arab Emirates; 2 Department of Mechanical and Aerospace Engineering, College of Engineering, United Arab Emirates University, Al Ain, United Arab Emirates; 3 Department of Automotive Engineering, Faculty of Engineering and Engineering Technology, Abubakar Tafawa Balewa University, Bauchi, Nigeria; 4 Institute of Public Health, College of Medicine and Health Sciences, United Arab Emirates University, Al Ain, United Arab Emirates; 5 Monash University Accident Research Centre, Monash University, Melbourne, VIC, Australia

**Keywords:** child passenger safety, injury severity, risk factors, road traffic crashes, UAE

## Abstract

Road traffic crashes (RTCs) are the leading cause of injuries and fatalities for children in the United Arab Emirates (UAE), with vehicle occupants representing the majority of child traffic casualties. However, despite the high incidence and severity of child occupant injuries in the UAE, no dedicated study has yet focused on the factors associated with injury risk for child occupants. All previous studies have focused on child occupants in Al Ain city and were conducted between 10 and 30 years ago. Accordingly, the current study sought to assess the current state of child occupant safety in the UAE using a recent national dataset for children aged 0–17 years. A retrospective, population-based analysis of UAE national crash data from 2012 to 2023 was conducted to identify key risk factors for child occupant injury severity. The principles of the safe system approach (SSA) were used to anchor the analysis of the identified risk factors. A hierarchical approach was employed where the initial model comprised the safe road users variables only. Variables corresponding to the other pillars of the SSA were added sequentially to evaluate the contribution of each SSA pillar to child passenger injury severity. Safe road user factors (gender, seating position, child age), safe speeds (posted speed), safe roads (road condition, lighting condition), and system management (emirate) were significantly associated with the severity of child occupant injuries. Strict enforcement of existing legislation regarding underage driving and front-row seating for young children, posted speed limit reductions, road safety behavioral change interventions targeting boys, child occupant safety awareness campaigns, and increased emergency response should be pursued to enhance child occupant safety in the UAE.

## 1 Introduction

The Safe System approach (SSA) to road safety aims to eliminate severe and fatal injuries from road traffic collisions (RTCs) by 2050 (1, 2). This is to be achieved by creating a road system that ensures no severe or fatal injuries are sustained in RTCs. The SSA is based on acknowledging human vulnerability and on designing a forgiving road system that focuses on interactions among safe speeds, safe road users, safe vehicles, safe roads, post-crash care, and system management (Figure 1). This holistic approach assigns responsibility for preventing severe and fatal injuries to all stakeholders involved in the road transport system (3–5).

**Figure 1:**
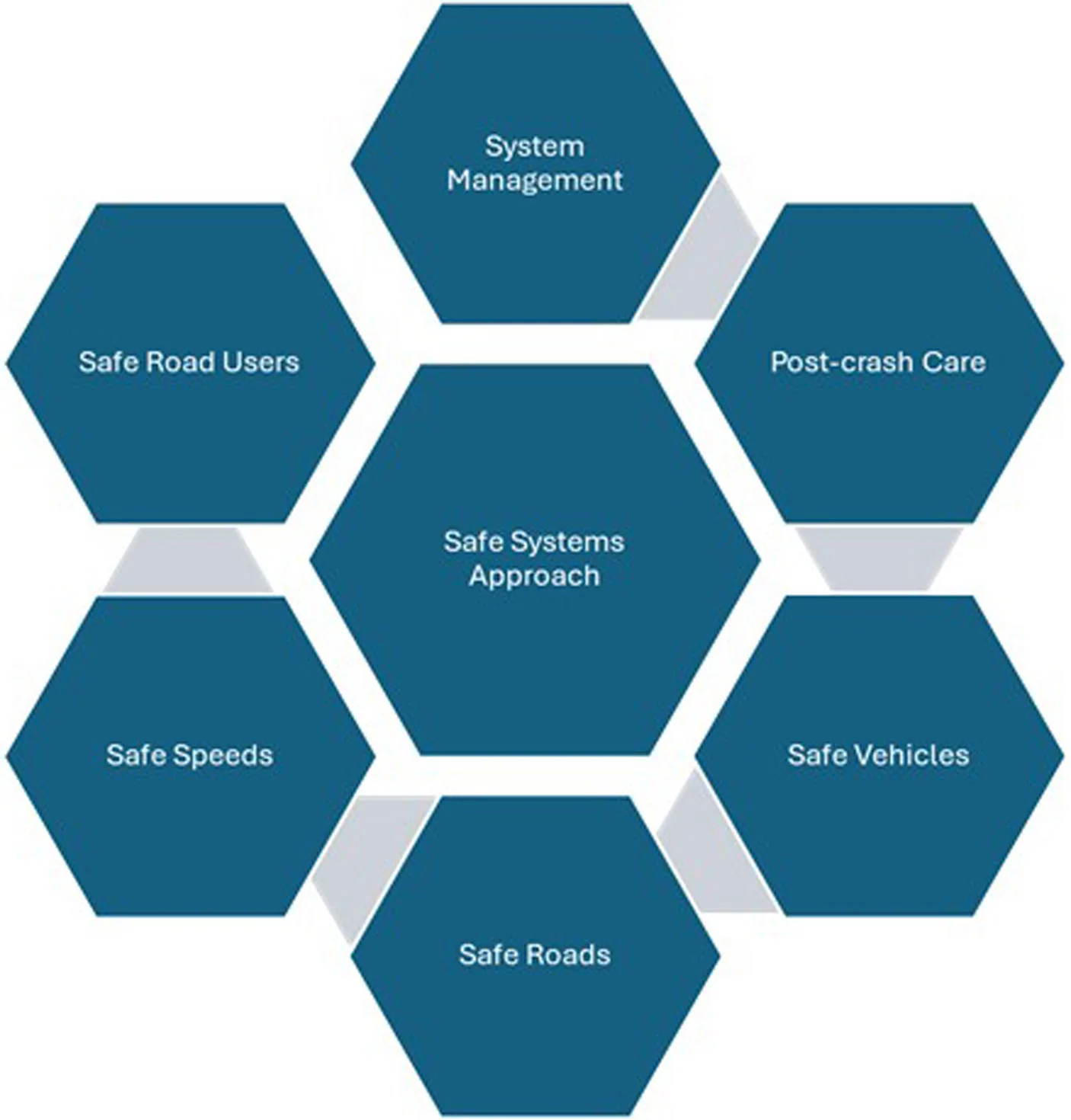
Safe system approach principles.

RTCs are the leading cause of death and serious injuries for children in the United Arab Emirates (UAE), with two-thirds of all child mortalities in the UAE due to RTCs (6). The majority of road traffic injuries (RTIs) occur among vehicle occupants in the UAE (7), with approximately 5,000 injuries recorded annually for child occupants aged 0–19 years, resulting in over $397 million in associated costs of these injuries (Figure 2). Despite this very high burden of child passenger RTIs, the literature on child passenger safety in the UAE is extremely limited (7). The majority of the available studies to date have mostly focused on issues relating to child restraint system (CRS) practices rather than child occupant RTI outcomes (8–20).

**Figure 2:**
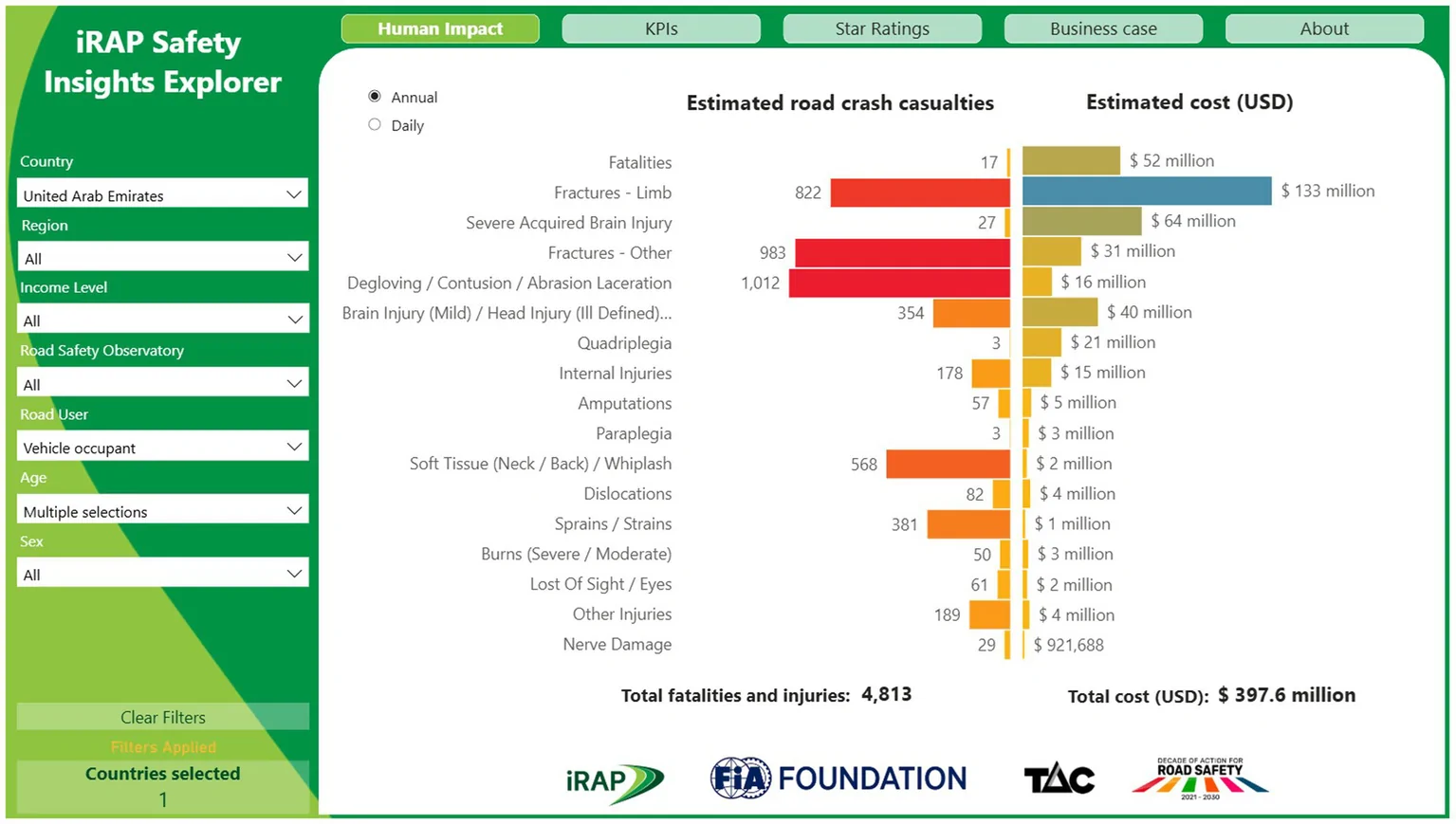
Burden of child occupant injuries for children aged 0–19 years in the UAE (source: international road assessment program safety insights explorer, https://irap.org/safety-insights-explorer/).

A comprehensive review of the literature revealed limited research on child occupant injuries and fatalities in the UAE (21–26). Almost all of the identified studies relied on data from Al Ain-based sources, namely–, Al Ain Department of Preventive Medicine, Al Ain Hospital, and Tawwam Hospital–, and were restricted to that city. Furthermore, most of these studies were conducted nearly 30 years ago, with the two most recent (conducted over a decade ago) relying on crash data that was already 10 years old at the time of analysis (24, 26). All these studies were either single or multiple hospital-based studies, used a prospective or retrospective study design, were exclusively focused on hospitals in Al Ain city, covered very small samples, and were limited to descriptive analyses.

While two recent studies have attempted to evaluate child passenger injury outcomes using a national dataset (27, 28), the dataset used in both studies was limited to the years 2012–2019. Additionally, one study focused on predicting injury severity (28) whereas the other was mostly descriptive (27) without detailed analyses regarding the factors associated with child occupant injury severity. Moreover, none of the identified studies was grounded on a conceptual framework. To address this significant gap, the current study aimed to identify key risk factors associated with child occupant injuries and fatalities using a recent national dataset (2012–2023) to provide recommendations for achieving comprehensive reductions in child occupant fatalities and injuries. The principles of the SSA were used to anchor the analysis of these risk factors. The variables in the dataset were conceptualized according to the different domains of the SSA including safe road users (child age, gender, and seating position), safe speeds (posted speed), safe roads (lighting condition and road condition), and system management (emirate). These factors have been previously reported to be associated with child occupant injury severity (27–35).

## 2 Materials and methods

### 2.1 Study design

This study was a retrospective population-based analysis of UAE national crash data from 2012 to 2023. The study was deemed exempt from ethical review under the UAE Ministry of Interior (MOI) Open Data Policy Charter (Supplementary File 1).

### 2.2 Data source

The data were obtained from the UAE MOI for the years 2012–2023. Complete national data was available starting from the year 2012 while the most recent data available at the time of analyses was for the year 2023 hence the selection of this study period. Data for Dubai Emirate was only available for the years 2022–2023 and therefore excluded from the analyses. The current study focused on child occupant injury severity and according to the United Nations Children’s Fund, individuals younger than 18 years are considered as children (36). Accordingly, child occupants aged 17 years or younger at the time of the crash were selected for inclusion in the analyses.

### 2.3 Child occupant demographics

Child occupant gender and emirate of residence were recorded as categorical variables while child occupant age was recorded as a continuous/numeric variable (Table 1).

**Table 1:** Dataset variables descriptions.

Category	Variable	Values
Safe road users	Gender^†^	Boy, girl
	Age^§^	0–17
	Seating position^†^	Driver, front, rear
Safe speeds	Posted speed^§^	10–160
Safe roads	Lighting condition^†^	Day, night enough lighting, night no/poor lighting
	Road condition^†^	Clear, not clear
System management	Emirate^†^	Abu Dhabi, Northern Emirates
Outcome	Injury severity^*^	Minor, moderate, severe, fatal

^†^Categorical variables. ^§^Numerical variables. *Outcome variable.

### 2.4 Crash attributes

Child occupant seating position was recorded as a categorical variable while posted speed limit was recorded as a numeric variable. It should be noted that 20% of the child occupants in the current study were seated on the driver seating position at the time of the crash. This indicates a very high rate of underage and unlicensed driving given that the legal driving age in the UAE is 18 years (37). Previous studies have reported this unsafe road safety habit (7, 24, 26–28, 38), and the issue seems to have persisted despite several interventions aimed at curtailing it (39).

### 2.5 Environmental conditions

Lighting condition and road condition were recorded as categorical variables.

### 2.6 Outcome

The outcome variable was injury severity, which was categorized into four levels according to the UAE MOI classification as follows:

- Minor: Injuries that required self-care or basic medical intervention.
- Moderate: Injuries that required medical intervention or a hospital stay.
- Severe: Injuries that required immediate medical intervention.
- Fatal: Injuries that led to death within 30 days of the crash (28, 40).

### 2.7 Data analyses

Descriptive statistical analyses were performed to describe the safe road users, safe speeds, safe roads, system management, and outcome variables. Frequencies and percentages were reported for categorical variables, while median and interquartile range were used to summarize numeric variables. Associations between categorical variables and child occupant injury severity were explored using Pearson’s Chi-squared test, while the relationship between numeric variables and child occupant injury severity was evaluated using the Kruskal-Wallis rank sum test. As indicated in the introduction section, the SSA principles were used to underpin the current study. Accordingly, variables were selected *a priori* based on the SSA framework. Safe road users (child age, gender, and seating position), safe speeds (posted speed), safe roads (lighting condition and road condition), and system management (emirate) were included in the multivariable models. A hierarchical approach was employed where the initial model comprised the safe road users variables only. Variables corresponding to the other pillars of the SSA were added sequentially to evaluate the contribution of each SSA pillar to child passenger injury severity. Since injury severity is ordinal (minor, moderate, severe, fatal), the proportional odds assumption was checked using the Brant test. While some variables satisfied the proportional odds assumption, others violated it. Accordingly, we fitted a partial proportional odds model whereby variables that satisfied the proportional odds assumption were modeled with constant proportional odds while those that violated it were modeled with partial proportional odds. The potential for multicollinearity among the variables was assessed using the Variance Inflation Factor (VIF), and all VIFs were < 2 for all the models. A significance level of 5% was used for all inferential statistics. All the statistical analyses were performed using R version 4.4.3.

## 3 Results

A total of 4,795 injured child occupants were included in the current study, of which 376 (7.8%) were severely injured, and 252 (5.3%) sustained fatal injuries (Table 2). The majority of the injured child occupants in this study were boys (69%), and their median age was 12 years (IQR: 6–16). Crashes that occurred in Abu Dhabi emirate accounted for the highest proportion of child occupant injuries (61%). Most injured child occupants were seated in the rear seat (60%), while the median posted speed limit was 60 km/h (IQR: 60–100). The majority of the child occupant crash injuries occurred under clear road conditions (88.9%) and in daylight conditions (53.7%). Figure 3 presents the trend of child occupant injuries between 2012 and 2023. The number of injuries sustained by child occupants decreased over the study period. The incidence of minor and moderate injuries also followed the same trend as that of the total injuries whereas severe and fatal injuries showed no change over the study period. The results for the safe road users model indicates that each additional year of age was associated with a 4% decrease in the odds of sustaining a more severe injury (moderate/severe/fatal) compared to minor injury (OR = 0.96, 95% CI: 0.92–0.99) (Tables 3). Conversely, each additional year of age was associated with a 10% increase in the odds of sustaining a more severe injury (severe/fatal) compared to minor/moderate injury (OR = 1.10, 95% CI: 1.07–1.13).

**Table 2:** Characteristics of variables in the study.

Characteristic	Minor, (*N* = 2,321; 48.4%)	Moderate, (*N* = 1,846; 38.5%)	Severe, (*N* = 376; 7.8%)	Fatal, (*N* = 252; 5.3%)	*p*-value
Emirate					**<0.001**
Abu Dhabi	1,324 (57.0%)	1,241 (67.0%)	221 (59.0%)	137 (54.0%)	
Northern Emirates	997 (43.0%)	605 (33.0%)	155 (41.0%)	115 (46.0%)	
Seating position					**<0.001**
Driver	329 (14.0%)	480 (26.0%)	112 (30.0%)	51 (20.0%)	
Front	406 (17.0%)	417 (23.0%)	53 (14.0%)	73 (29.0%)	
Rear	1,586 (69.0%)	949 (51.0%)	211 (56.0%)	128 (51.0%)	
Age, median (IQR)	11.0 (5.0, 15.0)	13.0 (8.0, 16.0)	14.0 (6.8, 16.0)	12.0 (4.8, 15.0)	**<0.001**
Gender					**<0.001**
Girls	830 (36.0%)	483 (26.0%)	88 (23.0%)	68 (27.0%)	
Boys	1,491 (64.0%)	1,363 (74.0%)	288 (77.0%)	184 (73.0%)	
Posted speed, median (IQR)	60.0 (60.0, 100.0)	60.0 (40.0, 100.0)	80.0 (60.0, 100.0)	80.0 (60.0, 113.0)	**<0.001**
Road condition					**0.005**
Clear	2,082 (90.0%)	1,627 (88.0%)	341 (91.0%)	211 (84.0%)	
Not clear	239 (10.0%)	219 (12.0%)	35 (9.0%)	41 (16.0%)	
Lighting condition					**<0.001**
Day	1,301 (56.0%)	961 (52.0%)	188 (50.0%)	127 (50.0%)	
Night enough lighting	905 (39.0%)	714 (39.0%)	148 (39.0%)	107 (42.0%)	
Night no/poor lighting	115 (5.0%)	171 (9.3%)	40 (11.0%)	18 (7.1%)	

Bold values denote *p* < 0.05.

**Table 3:** Safe road users.

Variable	Threshold	OR (95% CI)	*p*-value
Intercept	Constant	2.41 (2.20–2.65)	**<0.001**
Age	Minor vs. moderate/severe/fatal	0.96 (0.92–0.99)	**<0.001**
Age	Minor/moderate vs. severe/fatal	1.10 (1.07–1.13)	**<0.001**
Age	Minor/moderate/severe vs. fatal	1.20 (1.17–1.23)	**<0.001**
Gender			
Boys	Ref	Ref	Ref
Girls	Constant	0.79 (0.45–0.97)	**<0.001**
Seating position			
Driver	Ref	Ref	Ref
Front	Minor vs. moderate/severe/fatal	0.62 (0.54–0.72)	**<0.001**
Front	Minor/moderate vs. severe/fatal	1.05 (0.96–1.15)	0.309
Front	Minor/moderate/severe vs. fatal	0.66 (0.60–0.73)	**<0.001**
Rear	Minor vs. moderate/severe/fatal	0.53 (0.46–0.63)	**<0.001**
Rear	Minor/moderate vs. severe/fatal	0.60 (0.49–0.72)	**<0.001**
Rear	Minor/moderate/severe vs. fatal	0.63 (0.48–0.84)	**0.001**

AIC: 10157. Bold values denote *p* < 0.05.

**Figure 3:**
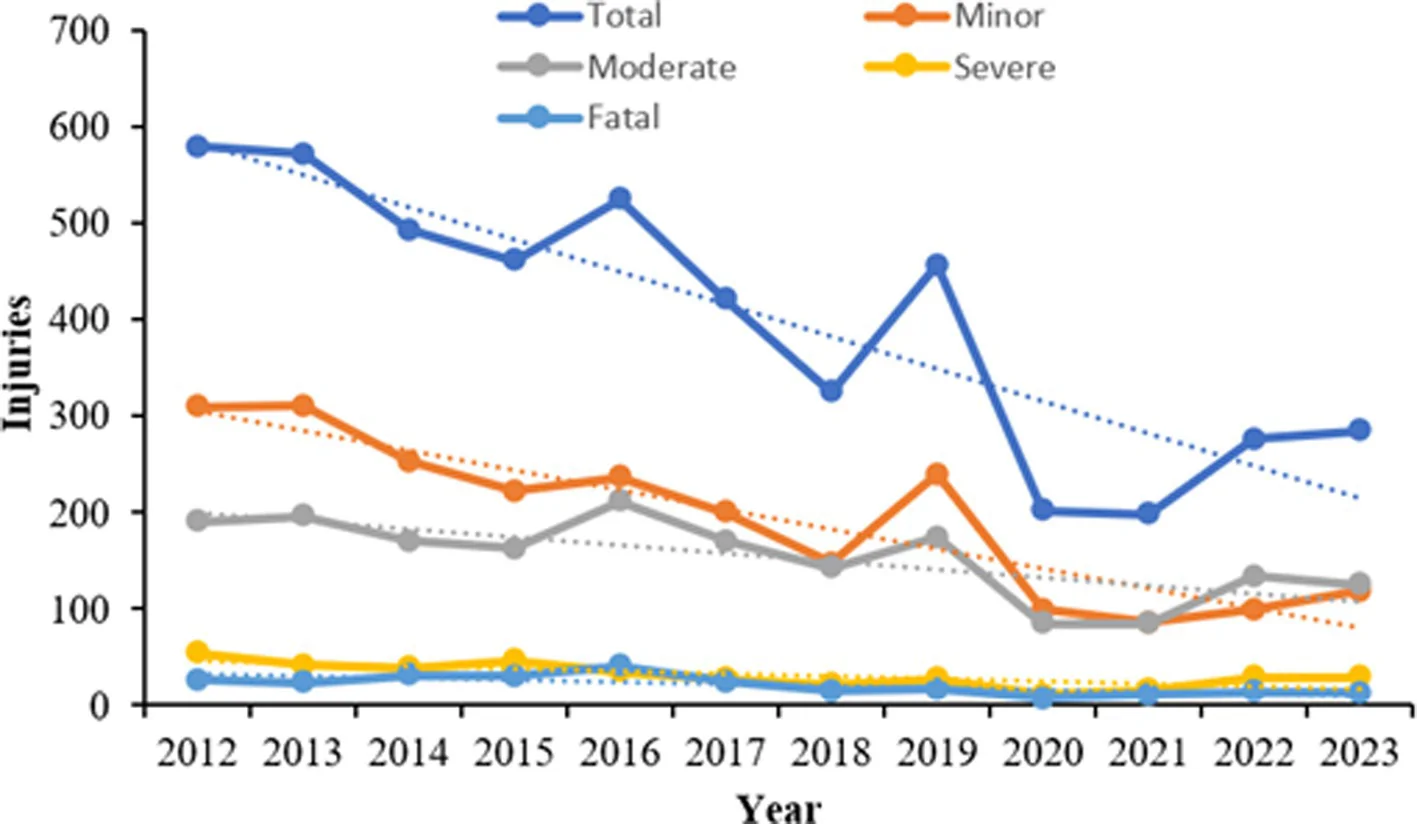
Child occupant injury severity trend.

Similarly, each additional year of age was associated with a 20% increase in the odds of sustaining a fatal injury (OR = 1.20, 95% CI: 1.17–1.23) compared to non-fatal injury (minor/moderate/severe). Girls had 21% lower odds of sustaining a more severe injury compared to boys (OR = 0.79, 95% CI: 0.45–0.97). Relative to child passengers seated in the driver seating position, child passengers seated in the front seating position had 38% lower odds of sustaining a more severe injury compared to minor injury (OR = 0.62, 95% CI: 0.54–0.72). Likewise, front seat child occupants had 34% lower odds of sustaining a fatal injury compared to non-fatal injury (OR = 0.66, 95% CI: 0.60–0.73). Compared to child passengers sitting in the driver seat, child occupants sitting in the rear seat had 47% lower odds of sustaining a more severe injury compared to minor injury (OR = 0.53, 95% CI: 0.46–0.63). Equally, child passengers seated in the rear seating position had 40% lower odds of sustaining a more severe injury compared to minor/moderate injury (OR = 0.60, 95% CI: 0.49–0.72). Additionally, rear seated child occupants had 37% lower odds of sustaining a fatal injury compared to non-fatal injury (OR = 0.63, 95% CI: 0.48–0.84). The effects of child passenger age, gender, and seating position decreased slightly but remained significant after accounting for posted speed. Child passengers sitting in the rear seat had 52% lower odds of sustaining a more severe injury compared to those sitting in the driver seat (OR = 0.48, 95% CI: 0.41–0.57).

While statistically significant, the effect of posted speed on the odds of sustaining a more severe injury for child occupants was very small (Tables 4). Road condition and roadway lighting condition were associated with child passenger injury severity (Tables 5). Child occupants involved in daylight crashes had 42% lower odds of sustaining a more severe injury compared to those involved in nighttime crashes with no or poor lighting condition (OR = 0.58, 95% CI: 0.19–0.97). Similarly, child passengers involved in nighttime crashes with enough lighting had 4% lower odds of sustaining a more severe injury (OR = 0.96, 95% CI: 0.64–0.99). The not clear road conditions were associated with an 18% lower odds of a more severe injury compared to minor injury for child occupants (OR = 0.82, 95% CI: 0.69–0.97). Likewise, the not clear road conditions were associated with a 20% lower odds of a fatal injury compared to non-fatal injury (OR = 0.80, 95% CI: 0.73–0.88) for child passengers. On the other hand, the not clear road conditions were associated with a 10% higher odds of a more severe injury (severe/fatal) compared to minor/moderate injury (OR = 1.10, 95% CI: 1.01–1.20) for child occupants. The effects of child passenger age, gender, seating position, and posted speed remained significant. Child passenger age, gender, seating position, posted speed, lighting condition, and road condition remained independently associated with child occupant injury severity after adjusting for all the SSA pillars (Tables 6). Child passengers in the Northern Emirates had 26% higher odds of a more severe injury (moderate/severe/fatal) compared to minor injury (OR = 1.26, 95% CI: 1.12–1.42).

**Table 4:** Safe speeds.

Variable	Threshold	OR (95% CI)	*p*-value
Intercept	Constant	4.01 (3.23–4.98)	**<0.001**
Age	Minor vs. moderate/severe/fatal	0.96 (0.92–0.97)	**<0.001**
Age	Minor/moderate vs. severe/fatal	1.07 (1.03–1.12)	**<0.001**
Age	Minor/moderate/severe vs. fatal	1.13 (1.08–1.18)	**<0.001**
Gender			
Boys	Ref	Ref	Ref
Girls	Constant	0.77 (0.68–0.88)	**<0.001**
Seating position			
Driver	Ref	Ref	Ref
Front	Minor vs. moderate/severe/fatal	0.59 (0.52–0.69)	**<0.001**
Front	Minor/moderate vs. severe/fatal	0.98 (0.89–1.08)	0.692
Front	Minor/moderate/severe vs. fatal	0.61 (0.55–0.68)	**<0.001**
Rear	Constant	0.48 (0.41–0.57)	**<0.001**
Posted speed	Minor vs. moderate/severe/fatal	0.98 (0.97–0.99)	**<0.001**
Posted speed	Minor/moderate vs. severe/fatal	1.02 (1.01–1.06)	**0.042**
Posted speed	Minor/moderate/severe vs. fatal	1.08 (1.05–1.12)	**<0.001**

AIC: 10122. Bold values denote *p* < 0.05.

**Table 5:** Safe roads.

Variable	Threshold	OR (95% CI)	*p*-value
Intercept	Constant	4.31 (3.95–4.71)	**<0.001**
Age	Minor vs. moderate/severe/fatal	0.97 (0.95–0.99)	**<0.001**
Age	Minor/moderate vs. severe/fatal	1.07 (1.04–1.10)	**<0.001**
Age	Minor/moderate/severe vs. fatal	1.14 (1.11–1.17)	**<0.001**
Gender			
Boys	Ref	Ref	Ref
Girls	Constant	0.79 (0.42–0.97)	**<0.001**
Seating position			
Driver	Ref	Ref	Ref
Front	Minor vs. moderate/severe/fatal	0.59 (0.51–0.68)	**<0.001**
Front	Minor/moderate vs. severe/fatal	0.97 (0.66–0.99)	**<0.001**
Front	Minor/moderate/severe vs. fatal	0.61 (0.16–0.92)	**<0.001**
Rear	Constant	0.48 (0.42–0.55)	**<0.001**
Posted speed	Minor vs. moderate/severe/fatal	0.98 (0.93–0.99)	**<0.001**
Posted speed	Minor/moderate vs. severe/fatal	1.01 (1.00–1.08)	**<0.001**
Posted speed	Minor/moderate/severe vs. fatal	1.04 (1.03–1.07)	**<0.001**
Lighting condition			
Night no/poor lighting	Ref	Ref	Ref
Night enough lighting	Constant	0.96 (0.64–0.99)	**<0.001**
Day	Constant	0.58 (0.19–0.97)	**<0.001**
Road condition			
Clear	Ref	Ref	Ref
Not clear	Minor vs. moderate/severe/fatal	0.82 (0.69–0.97)	**0.020**
Not clear	Minor/moderate vs. severe/fatal	1.10 (1.01–1.20)	**0.034**
Not clear	Minor/moderate/severe vs. fatal	0.80 (0.73–0.88)	**<0.001**

AIC: 10098. Bold values denote *p* < 0.05.

**Table 6:** System management.

Variable	Threshold	OR (95% CI)	*p*-value
Intercept	Constant	3.91 (3.38–4.52)	**<0.001**
Age	Minor vs. moderate/severe/fatal	0.96 (0.92–0.98)	**<0.001**
Age	Minor/moderate vs. severe/fatal	1.08 (1.06–1.12)	**<0.001**
Age	Minor/moderate/severe vs. fatal	1.14 (1.13–1.19)	**<0.001**
Gender			
Boys	Ref	Ref	Ref
Girls	Constant	0.79 (0.48–0.80)	**<0.001**
Seating position			
Driver	Ref	Ref	Ref
Front	Minor vs. moderate/severe/fatal	0.58 (0.51–0.67)	**<0.001**
Front	Minor/moderate vs. severe/fatal	0.98 (0.86–1.08)	0.692
Front	Minor/moderate/severe vs. fatal	0.61 (0.45–0.66)	**<0.001**
Rear	Constant	0.48 (0.42–0.56)	**<0.001**
Posted speed	Minor vs. moderate/severe/fatal	0.98 (0.95–0.99)	**<0.001**
Posted speed	Minor/moderate vs. severe/fatal	1.01 (1.00–1.04)	**<0.001**
Posted speed	Minor/moderate/severe vs. fatal	1.04 (1.02–1.06)	**<0.001**
Lighting condition			
Night no/poor lighting	Ref	Ref	Ref
Night enough lighting	Constant	0.95 (0.71–0.96)	**<0.001**
Day	Constant	0.59 (0.57–0.61)	**<0.001**
Road condition			
Clear	Ref	Ref	Ref
Not clear	Minor vs. moderate/severe/fatal	0.79 (0.66–0.94)	**0.008**
Not clear	Minor/moderate vs. severe/fatal	1.11 (1.00–1.23)	**0.045**
Not clear	Minor/moderate/severe vs. fatal	0.80 (0.72–0.88)	**<0.001**
Emirate			
Abu Dhabi	Ref	Ref	Ref
Northern Emirates	Minor vs. moderate/severe/fatal	1.26 (1.12–1.42)	**<0.001**
Northern Emirates	Minor/moderate vs. severe/fatal	1.02 (0.88–1.18)	0.801
Northern Emirates	Minor/moderate/severe vs. fatal	1.09 (0.95–1.26)	0.231

AIC: 10090. Bold values denote *p* < 0.05.

## 4 Discussion

This study identified a broad range of factors that influence crash injury severity among child occupants in the UAE. Consistent with the SSA, these factors span multiple domains, including road users, roadway posted speed, roads and roadsides, and the broader regulatory and organizational environment. Importantly, factors like child occupant seating position and roadway posted speed do not align exclusively with a single SSA pillar. Rather, they interact across multiple pillars. Consequently, the findings are presented within the SSA framework to aid interpretation and policy development; however, some factors should be recognized as cross-cutting and interconnected, requiring coordinated responses across multiple SSA pillars.

### 4.1 Safe road users

#### 4.1.1 Seating position

Child occupant seating position was associated with injury risk in the present study, with children seated in the driver seat having higher odds of injury compared to those in the front and rear seat. Front and rear seat child occupants had lower odds of progressing to a higher injury severity level compared to child occupants in the driver seat. This indicates that underaged and unlicensed drivers are extremely vulnerable to sustaining more severe injuries compared to child occupants seated in the front and rear seats. Factors such as steering wheel contact, driver airbag deployment, instrument panel contact, and frontal impact intrusion may contribute to the observed higher injury risk for underage drivers. The current findings are consistent with the literature on child occupant injuries, where vehicle seating position has been established to be associated with the severity of injuries sustained by child occupants (29, 30, 41). Our findings also align with the results of several previous studies, which showed the rear seat to be the safest sitting position for children (27, 28, 42–45).

Inappropriate/unsafe seating positions (front row seating) for child occupants younger than 13 years who are traveling unrestrained (for all child occupants) are more common among teenage drivers transporting children. They are associated with a higher injury risk for child occupants (46). Twenty percent (972) of the child occupants in the current study were drivers aged 17 years or younger at the time of the crash. This finding suggests that not only are they (teen drivers) at an increased risk of injury, but they are more likely to be transporting other children unrestrained and seated in the front row, thereby increasing their (unrestrained and front row child occupants) odds of sustaining injuries in crashes. Additionally, children aged less than 14 years sitting in the front seat of vehicles have been reported to be at increased risk of severe injuries in crash situations due to airbag deployment (41, 47–49). In the context of the SSA, vehicle seating position falls under the safe road users pillar because it is a choice made by the vehicle users whether to sit in the rear, front, or driver seat, thereby making it a road user behavioral issue as well as compliance with relevant legislative requirements regarding vehicle seating position.

The findings of the current study regarding the unsafe seating positions adopted by child occupants supports the safe road users principle regarding safe seating positions for child occupants. Therefore, strategies aimed at ensuring that parents and children in the UAE recognize the importance of safe vehicle seating positions should be employed through road user education programs and strict enforcement of the relevant UAE traffic safety legislations (especially those mandating rear seating for children less than 145 cm in height or younger than 10 years old and prohibiting driving for children younger than 18 years old) (37).

#### 4.1.2 Gender

Child occupant gender was associated with injury risk in the current study, with girls less likely to sustain injuries compared to boys. Boys are usually more likely to sustain crash injuries compared to girls due to behavioral differences, as girls are more sensitive to traffic and less likely to engage in risky behaviors in traffic environments (50, 51). Additionally, as discussed above, children sitting in the driver and front seats have increased risk of injury. In the current study, boys accounted for 95% (926) and 72% (680) of children seated in the driver and front seating positions, respectively. This may explain why girls had a lower injury risk than boys in the current study. Previous research exploring the effect of gender on child occupant injury risk has presented conflicting results. Although several studies have reported the association between gender and injury risk for child occupants (24, 26, 27, 52), other studies have reported no association between gender and injury risk for child occupants (28, 53–55).

While gender differences in adult occupant injury risk can be attributed to anatomical, physiological, and biomechanical differences between males and females (56–58), the gender difference in child occupant injury risk is mainly influenced by risk exposure, situational or behavioral factors, and social patterns rather than physiological or biomechanical fragility. Boys are more likely to be traveling unrestrained, driving underage, or riding in the front seat, thereby increasing their odds of sustaining injuries in crash situations (7, 24, 26, 27, 52). Gender falls under the safe road user pillar of the SSA because it pertains to road user characteristics. As discussed above, gender influences risk exposure and injury outcomes. Behavioral change interventions, educational training programs, and enforcement campaigns targeting boys should be adopted to reduce their risk of injuries as child occupants effectively.

#### 4.1.3 Age

The association between child occupant age and injury severity followed a heterogeneous pattern, varying across the different injury severity thresholds. Increasing child occupant age was associated with decreased odds of sustaining at least a moderate injury compared to minor injury. Beyond the minor injury threshold, increasing age was associated with higher odds of sustaining a severe or fatal injury compared to minor or moderate injury as well as higher odds of sustaining a fatal injury compared to non-fatal injury among child occupants. Factors relating to increased crash tolerance, larger body mass, skeletal maturity, and enhanced musculature may explain the finding that increasing child occupant age was associated with lower odds of sustaining moderate or higher severity injury compared to minor injuries. Older children are more likely to be restrained using the vehicle seatbelt thereby reducing their susceptibility to lower severity injuries especially in low impact crashes (59).

On the other hand, increasing age was associated with higher likelihood of sustaining severe or fatal injury possibly due to the differences in the mechanisms for higher and lower severity injuries. Older children are usually more likely to travel in inappropriate seating positions, are more exposed to high speed travel, are involved in higher severity crashes, and transition prematurely from CRS to adult seatbelt. These unsafe practices often result in severe thoracic and abdominal injuries in high impact crashes due to seatbelt loading (60). The current findings regarding the association between child occupant age and injury severity aligns with the safe road users pillar of the SSA. Accordingly, child occupant protection should reflect the different stages of child development through appropriate CRS selection and age-appropriate transition from one form of CRS to the next. Additionally, targeted campaigns aimed at reducing risky child occupant behaviors, raising awareness regarding unsafe child occupant behaviors, and educational/enforcement interventions geared toward promoting safer child occupant habits should be pursued to enhance child passenger safety in the UAE.

### 4.2 Safe speeds

#### 4.2.1 Posted speed

Roadway speed limits have been identified as a significant factor in increased injury risk for children (61). The association between posted speed and child occupant injury severity varied across the injury severity thresholds in the present study. Higher posted speeds were associated with a marginally lower odds of sustaining injury beyond the minor severity threshold. However, beyond the minor severity threshold, higher posted speeds were associated with slightly higher odds of sustaining severe or fatal injury compared to minor or moderate injury and higher odds of fatal injury compared to non-fatal injury. Possible explanations for these counterintuitive patterns includes the difference between posted speed and impact/travel speed. Posted speeds generally represent roadway environmental characteristic rather than travel or impact speed. Roads with higher posted speeds usually have better pavement quality, wider lanes, median separation, high design standards, controlled access, etc. These road design features can reduce the likelihood of sustaining moderate severity injuries despite the high posted speeds. Therefore, the protective association between posted speed and the first injury severity threshold may reflect the effect of high posted speed road’s functional classification and road design features rather than the independent effect of speed. On the other hand, the high kinetic energy due to higher posted speeds increases the odds of severe or fatal injuries in the event of a crash.

Another plausible explanation could be the direction of association of different injury causation mechanisms across the severity thresholds. While the first injury severity threshold is mostly associated with factors related to the occurrence of injury beyond the minor threshold, the higher severity threshold are mostly affected by factors like impact energy, child occupant biomechanics, child restraint status, etc. It should be noted that while the observed increase in the odds of sustaining severe or fatal injury for child occupants in the current study per unit increase in posted speed was small, these odds become substantial over larger increases in posted speed (for instance 25 to 50 km/h). This indicates that the failure to maintain safe speeds affects injury severity of child occupants. Speeding has been reported to be associated with more severe injuries for child occupants in several studies (27, 28, 53, 54, 62, 63). Numerous studies have shown that the majority of severe and fatal injuries in the UAE occur on roads with high posted speed limits (80–160 km/h) (38, 64, 65). While 49% of child occupant injuries in the present study were sustained on roads with posted limits of 80–160 km/h, 53% of severe and 64% of fatal injuries occurred on those roads. This shows the adverse impact of speed on the injury severity of child occupants and reinforces the safe speeds pillar of the SSA as well as the complementary nature of the different pillars of the SSA (in this case safe speeds and safe roads).

A key pillar of the SSA is safe speeds, and it has been reported that a 1% increase in average speed results in a 4–5 and 3% increases in the risks of fatal and severe injuries, respectively (66). On the other hand, a 5% reduction in average speed results in a 30% decrease in the risk of fatal injury (67). Several international organizations including the United Nations, the World Bank, the World Health Organization, and the International Transport Forum, have proposed safe speed (survivable impact speed) limits based on the traffic volume, road environment, and the likelihood of crashes (2, 5, 66). While safe speed limits have been computed for all road users, vulnerable road users (children and older adults) have been identified to be at increased risk even at these safe speeds (67). Based on the foregoing, it is recommended that traffic safety authorities in the UAE should consider revising the posted speed limits on the road network in the country to reduce the impact energy in crash situations by accounting for human tolerance to impact forces. This should be done by ensuring that posted speed limits on the road network are within the recommended safe speed limits for the road type, road user type, traffic volume, road function, road environment, and crash potential. This will ensure improved RTI outcomes for child occupants and all other road users in the country. Additionally, public awareness campaigns regarding the dangers of speeding and the benefits of adhering to safe speed limits should be prioritized.

### 4.3 Safe roads

#### 4.3.1 Road condition

In the safe systems framework, the safe roads pillar focuses on designing road infrastructure that either reduces the severity of crashes or prevent them altogether. Data regarding road condition helps traffic safety authorities to identify infrastructure issues and high-risk locations on the road network. This is useful in developing countermeasures for enhancing the road network to make it safer. Road design factors are one of the possible reasons for the severity of crashes for a particular road condition. The findings of the present study indicated that clear road conditions posed the greatest injury risk for child occupants. The association between road condition and injury severity varied across the different injury severity thresholds. Crashes that occurred on the not clear (obstacle, curved, pothole, blocked, crowded, narrow) road conditions were associated with reduced odds of sustaining injuries beyond the minor injury severity level. Conversely, these road conditions were associated with higher odds of sustaining severe or fatal injury compared to minor or moderate injury. On the contrary, they were associated with decreased odds of sustaining fatal injury compared to non-fatal injury.

A possible explanation for this finding is that drivers usually reduce their speed on roads with challenging surface, operating, or geometric characteristics (narrow, curved, crowded, and other deteriorated road conditions) due to driving difficulties on these road conditions (68). This result in reduced impact energy thereby leading to lower likelihood of fatal injury and higher odds of minor injury. On the other hand, factors such as obstacles, narrow width, crowdedness, and curviness on these road conditions may increase the severity of child occupant injuries from moderate to severe threshold. It should be noted that the not clear road conditions combines roadway characteristics with several distinct features which may affect injury severity through different mechanisms. Therefore, the variation in the direction of its association across the injury severity thresholds could be due to the fact that its estimated effect correspond to the average influence of a diverse group of road conditions. The findings of the current study reinforces the safe roads principle of the SSA and indicates the need for improvements in roadway design such as widening narrow road sections and enhanced curve design to reduce child occupant injury risk. Additionally, roadway maintenance, improved traffic flow, and geometric enhancements should be prioritized to protect child occupants and other road users.

#### 4.3.2 Lighting condition

Daylight and well-lit nighttime lighting conditions were associated with lower odds of sustaining more severe injuries for child occupants compared to nighttime inadequate lighting conditions. These findings are consistent with previous studies regarding the association between roadway lighting condition and crash injury severity (69–72). Insufficient roadway lighting result in poor driver risk perception, delayed hazard response, delayed braking, higher impact speeds, and impaired visibility thereby increasing crash severity. Improvements in roadway lighting infrastructure are effective in improving driver visibility and reduce injury crashes.

Roadway lighting condition falls under the safe roads pillar of the SSA. The findings of the present study supports the safe roads principle regarding the design of roadway infrastructure to accommodate human limitations such as impaired visibility during nighttime driving. While poor or no nighttime lighting represented the least forgiving roadway lighting condition for child occupants, well-lit nighttime and daytime lighting conditions were associated with reduced injury severity. This suggests that adequate roadway lighting conditions can mitigate child occupant injury severity. Therefore, roadway lighting improvements should be undertaken particularly on roads with regular child occupant travel, high traffic volumes, and high posted speeds to reduce the risk of child occupant injuries in the UAE.

### 4.4 System management

#### 4.4.1 Emirate

Child occupants in the Northern Emirates were more likely to sustain injuries beyond the minor injury severity threshold compared to those in Abu Dhabi emirate. However, there was no difference in severe and fatal injury odds between child occupants in Abu Dhabi emirate and the Northern Emirates. These findings suggest that the association between emirate and injury severity is only present at the minor injury severity threshold but not evident at the higher severity thresholds. Crashes in the Northern Emirates resulted in higher risk of injuries beyond the minor injury severity threshold for child occupants in the present study. We postulate that crashes occurring in the Northern Emirates (especially rural or remote locations) will have longer emergency response times thereby increasing the severity of injuries sustained by child occupants. Additionally, access to trauma care could be limited at certain remote locations. The findings of the current study regarding the high-risk emirates for child occupants should be employed to optimize the placement of emergency resources like trauma centers or ambulance stations at the Northern Emirates. Emirate best fits under the system management pillar of the SSA because it serves as an essential data element that helps in understanding system performance and guiding improvements across all the other pillars.

Data regarding the emirates provides information regarding the trends, risk factors, and patterns of crashes at the different emirates of the country. These data are useful in informing safety interventions (road maintenance, awareness campaigns, etc.), policy, and strategic planning targeted toward improving the entire safe system. Emirate data is also useful in analyzing crash patterns and risk factors and aids in the design of tailored interventions. It is a critical element that influence the effective delivery of post-crash care. Factors including limited trauma care infrastructure, delayed emergency care response, and longer distances to definitive care can contribute to higher injury risk for child occupants at certain locations (73–76). The findings of the current study regarding crash-prone emirate (Abu Dhabi) as well as the emirates associated with increased injury risk (Northern Emirates) should serve as a basis for data-driven safety interventions tailored toward reducing the incidence and severity of child occupant injuries at all the emirates in the UAE. Figure 4 presents the interplay between the seven factors discussed above and the different pillars of the SSA.

**Figure 4:**
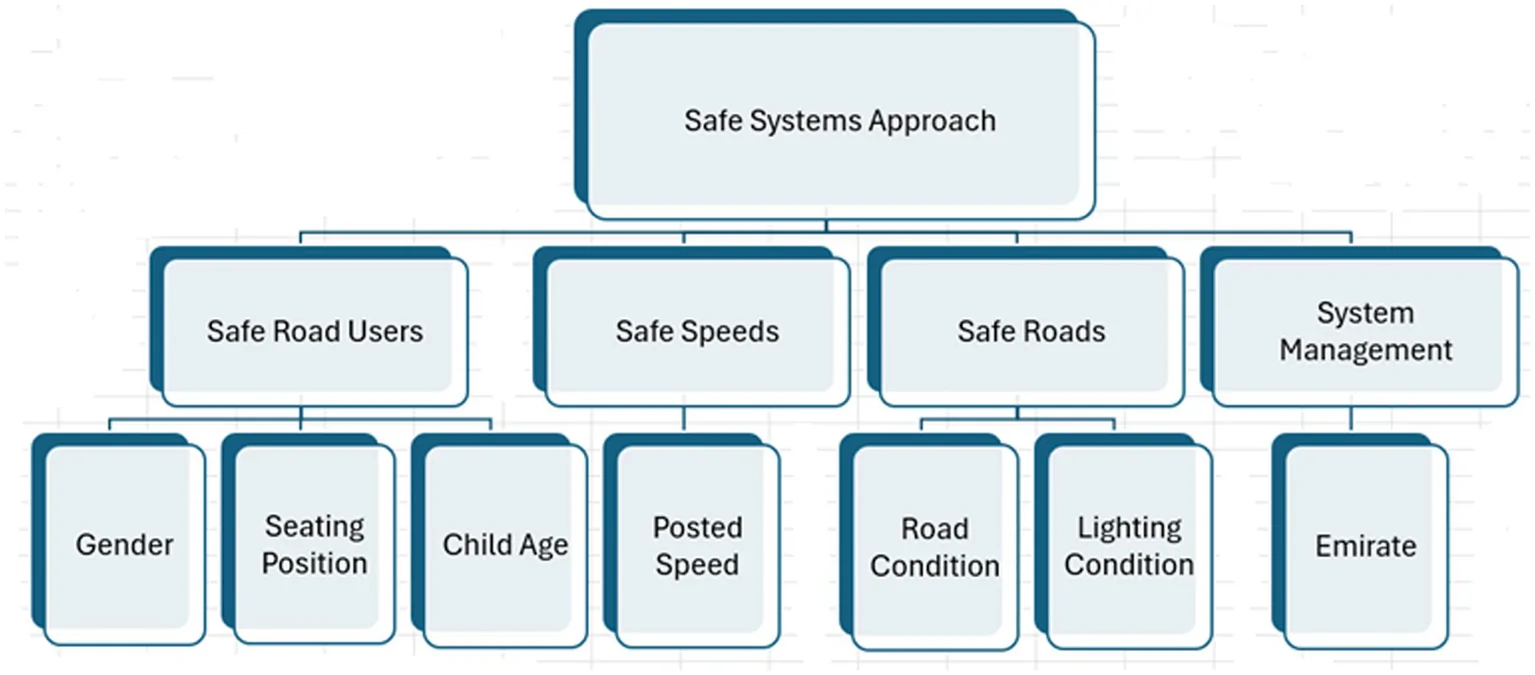
Factors affecting child occupant injury based on the safe system approach.

It would have been useful to provide comparisons with regional literature (studies from Gulf countries) due to similar traffic environments to improve the contextualization of the findings of the current study. However, like the case with UAE child passenger research, majority of the studies from the Gulf region on child passenger safety had mostly focused on child restraint patterns rather than injury outcomes (77–82). Very few studies were identified on child RTI outcomes. These studies were exclusively hospital-based and focused on general pediatric trauma (83–85), general RTI patterns (86), and pediatric RTC trauma (87–90) rather than vehicle occupant crash injury severity analyses. No national-level study was identified in the literature making direct comparison with the current study difficult. Nevertheless, similar findings to the findings of the current study were reported regarding underage driving and unsafe front sitting among child occupants (87, 88). This highlights the regional prevalence of these unsafe seating positions adopted by child occupants and the need for a concerted regional effort to address these unsafe child occupant transportation patterns.

## 5 Limitations

The present study did not include child occupant crash injury data for Dubai emirate due to the unavailability of that data for 10 out of the 12 years of the study period. This exclusion could affect the generalizability of the current study’s findings due to the differences in population characteristics, traffic volume, and injury patterns between Dubai and the other emirates. Despite this limitation, the current study presents an assessment of child passenger injury severity for the remaining six emirates of the UAE over a 12-year period thereby providing important insights. Data on the restraint status of the child occupant and vehicle type were available only for Abu Dhabi emirate and were missing for all other emirates in the dataset. Accordingly, we excluded them from the analyses in the current study. However, these factors have been established to have a very strong association with child occupant injury severity (26, 91–94). While data regarding gender-related behavioral characteristics and traffic exposure were also missing in the dataset, vehicle seating position data was available. This can serve as a proxy for crash injury exposure inside the vehicle and partly explains the gender differences in child occupant injury severity as highlighted in the discussion section. These limitations should be considered while interpreting the findings of the present study. Future studies should incorporate key variables like child restraint status, vehicle type, behavioral characteristics, traffic exposure, and a nationally representative RTI data to provide a more comprehensive evaluation of child occupant crash injury outcomes.

## 6 Conclusion

The findings of the current study have several policy and practice implications. First, strategies aimed at ensuring that parents and children in the UAE recognize the importance of safe vehicle seating positions should be employed through road user education programs and strict enforcement of the relevant UAE traffic safety legislations (especially those mandating rear seating for children less than 145 cm in height or younger than 10 years old and prohibiting driving for children younger than 18 years old). Second, behavioral change interventions, educational training programs, and enforcement campaigns targeting boys should be adopted to effectively reduce their risk of injuries as child occupants. Third, child occupant protection should reflect the different stages of child development through appropriate CRS selection and age-appropriate transition from one form of CRS to the next. Additionally, targeted campaigns aimed at reducing risky child occupant behaviors, raising awareness regarding unsafe child occupant behaviors, and educational/enforcement interventions geared toward promoting safer child occupant habits should be pursued to enhance child passenger safety in the UAE. Fourth, traffic safety authorities in the UAE should consider revising the posted speed limits on the road network in the country to reduce the impact energy in crash situations by accounting for human tolerance to impact forces. This should be done by ensuring that the posted speed limits on the road network are within the recommended safe speed limits based on the road type, road user type, traffic volume, function of the road, road environment, and possibility of crashes. These will ensure enhanced RTI outcomes for child occupants as well as all other road users in the country. Additionally, public awareness campaigns regarding the dangers of speeding and the benefits of adhering to safe speed limits should be prioritized. Fifth, there is a need for improvements in roadway design such as widening narrow road sections and enhanced curve design to reduce child occupant injury risk. Additionally, roadway maintenance, improved traffic flow, and geometric enhancements should be prioritized to protect child occupants and other road users. Sixth, roadway lighting improvements should be undertaken particularly on roads with regular child occupant travel, high traffic volumes, and high posted speeds to reduce the risk of child occupant injuries in the UAE. Finally, the findings of the current study regarding crash-prone emirate (Abu Dhabi) as well as the emirates associated with increased injury risk (Northern Emirates) should serve as a basis for data-driven safety interventions tailored toward reducing the incidence and severity of child occupant injuries at all the emirates in the UAE. Ultimately, these improvements will contribute to significantly better safety outcomes and increase the chances of survival for child occupants involved in traffic incidents.

## Data Availability

Publicly available datasets were analyzed in this study. This data can be found at: https://moi.gov.ae/ar/open.data.aspx.
