# Supplementary material for: Child occupant injuries in the United Arab Emirates: a case for a safe system approach
Source: Front Public Health. 2026 Sep 25;14:1844331. doi: 10.3389/fpubh.2026.1844331 (PMC13650741; doi:10.3389/fpubh.2026.1844331)
Supplement: Supplementary file 1 [file Supplementary_file_1.pdf]

Open Data Policy Charter

Through the Department of Policies, the Ministry of Interior developed various mechanisms for making general or institutional policies or "Policies Charter." This is done in accordance with government requirements and international trends in making institutional and general policies. Adherence to the contents of this charter will have a significant effect on the Ministry of Interior, thus promoting performance, excellence and institutional flexibility and qualifying the Ministry for competitiveness in terms of workplace system at the federal government level as well as local and international levels.

First: Objective of Policy Charter

The Policy Charter aims to set forth the responsibilities of the Ministry of Interior and the public regarding open data and the mechanism for its use. By definition, open data is referred to as data that anyone can use freely without technical or financial restrictions, in addition to reusing or publishing it.

Second: Duties and Responsibilities of Policy Charter

Ministry of Interior's responsibilities:

- The Ministry posts data on its electronic portal so that users can access transparently and freely.
- Providing data sustainably.
- Giving easy and flexible access to data.
- Publishing data in usable file formats.
- Enabling end-users to express their opinion on the open data available on the website.
- Providing accurate description of the size and formats of the available files and indicating date of publication.

Responsibilities of the public:

- When using this data, it must be indicated that its source is the Ministry of Interior, in order to protect the Ministry's ownership of the data, its credibility and the validity of its source, while mentioning the name of the file and the date of its publication.
- A data user must not misrepresent the data or its source or mislead others.
- Data should not be used for political purposes or to support illegal or criminal activity or be used in racist or discriminatory comments or for the purpose of instigation or to negatively undermine culture or equality. It must not be used in incitement or any illegal activity or contrary to the values of the UAE society.
- The Ministry of Interior is not responsible under any circumstances for any direct or indirect, incidental or ancillary, special or exceptional damage which may arise from the use or inability to use this open data, including without limitation loss of income, loss of reputation, loss of work, loss of data, computer breakdown or wrong operation, or any other damage.
- A user of open data on the Ministry of Interior's website acknowledges that he/she shall use open data at his/her own responsibility, and the Ministry shall not have any responsibility whatsoever to update all open data in the future, especially when it is based on current assumptions and predictions that may lead to the occurrence of some risks and raise some doubts over the discrepancy between actual results and the foreseen future and the estimations thereof.
- The Ministry shall not be legally responsible for or compensate for any damage which the user or any other person may suffer as a result of breaching the confidentiality of information which the user may have transferred from the open data section.

Third: Policy Charter Scope

The scope of this Policy Charter shall include all organizational units across the Ministry of Interior

Reproduced with permission from "Open Data Policy Charter" United Arab Emirates Ministry of Interior.
